# Supplementary material for: Latent classes of substance use and delinquency in a Swedish national sample of adolescents and associated risk factors
Source: PLoS One. 2025 May 2;20(5):e0322515. doi: 10.1371/journal.pone.0322515 (PMC12047767; doi:10.1371/journal.pone.0322515)
Supplement: S1 Table — (DOCX) [file pone.0322515.s001.docx]

SUPPLEMENTAL MATERIAL ONLINE

| *S1 Table. Correlation matrix of indicator variables* | | | | | | |
| --- | --- | --- | --- | --- | --- | --- |
|  | Violent crime | Property crime | Theft | Alcohol | Cannabis | Other drugs |
| Violent crime | -- | 0.44 | 0.39 | 0.16 | 0.22 | 0.28 |
| Property crime |  | -- | 0.60 | 0.16 | 0.21 | 0.33 |
| Theft |  |  | -- | 0.23 | 0.27 | 0.29 |
| Alcohol |  |  |  | -- | 0.27 | 0.18 |
| Cannabis |  |  |  |  | -- | 0.58 |
| Other drugs |  |  |  |  |  | -- |
| *Note: All correlations were significant at p<0.05.* | | | | | | |
