# Supplementary material for: Latent classes of substance use and delinquency in a Swedish national sample of adolescents and associated risk factors
Source: PLoS One. 2025 May 2;20(5):e0322515. doi: 10.1371/journal.pone.0322515 (PMC12047767; doi:10.1371/journal.pone.0322515)
Supplement: Attachment — Submitted filename: Review 2024-11-28.docx [file pone.0322515.s002.docx]

Review PONE-D-24-39038

Overall comments. Big sample, suitable methodological choice for the aim, good to focus on SU and criminal offending in synergy, and an important research question (including current relevance). However, there are some issues regarding lack of information on the procedure, definition of groups and interpretation of results that need to be resolved before being able to decide on the study’s contribution to the field (i.e. above all information regarding methodology, see comments below).

Abstract: Regarding Background, clarify whether the aim was to identify risk factors associated *with later in adolescence belonging to specific* latent classes of SU and criminal offending or general risk factors for belonging to any “troubled” class. The four classes obtained represent all possible combinations. Results point to generalized risk factors for any class (including low/abstainers) according to Abstract, or is it only problem classes that is referred to? Which are the “less frequent behaviors” that is referred to in the Abstract? General recommendation regarding the abstract, either choose the most influential factors to present or lift the presentation to a more abstract level (away from specific results), and present it in a more overarching manner. Perhaps also a societal/clinical implication can be mentioned?

How did they know this risk factor preceded the class adherence (i.e. whether class adherence did not precede the risk factor), and not that the class adherence was already there by the first wave? Or have I misunderstood something basic within the design?

Regarding the Aim, a bit of specification would make it clearer: “Thus, the aim of the present study was to identify latent classes of substance use and criminal offending in a national representative Swedish sample and to identify developmental risk factors for each latent class.” Was the aim to identify *differentiating* developmental risk factors or *general* developmental risk factors for all “troubled” latent classes? (Again, I wonder if the developmental risk factors were associated with some kind of temporal assumption, that they preceded the latent class association or that they just were found for this specific/these specific classes?)

Material/method

Regarding participants, could percentages be provided for how many responded to the follow-up? What does “missing” entail here (male/female/missing)? The approx. 1500 participants who did not respond to T2, was any kind of non-response analysis conducted (regarding participant characteristics)? Can the final sample’s characteristics be considered to mirror also the non-responders? I have the same question regarding non-responders for the schools included, did the non-responders differ regarding important characteristics (e.g. SES-status of the area, size of school, rural/town etc)? This issue is touched upon briefly in the Limitations-section but should be considered more in the Discussion.

Material:

I have some questions regarding groups/categories used:

1) Was no standardized form regarding drugs used here? E.g. Dudit?

2) What was the rationale for including “illegally carrying a weapon” into violent crime?

3) what does the various total scores stand for – does it refer to some kind of ordinal scale numbers/categories? If so, what do the response options denote?

4) This is something that I personally is very interested in and I think could make this manuscript particularly informative: divide SU into alcohol; cannabis; stimulants; sedatives. Since sensation-seeking is investigated, there is a good basis in research to see if crime is particularly related to stimulants rather than sedatives among adolescents. Or check for prevalences/percentages of various drug categories within the respective latent classes. This comment of mine is not necessary to do something about per se, but I think it could give this manuscript an edge and inform the field in an important way. But I understand that group comparisons are difficult due to the power issue.

Regarding the comment that “Some have suggested that correlations greater than 0.5 among indicators might create statistical problems with multicollinearity [29]. However, these indicators were deemed theoretically important and in line with the objective of the study and therefore included.”, it would help the reader if the author clearly notes here what kind of interpretation error that this choice could have resulted in within this study.

Could you explain a little more to the reader/provide a rational in the text regarding the following analytic steps: To see what factors at T1 predicted classes at T2, pairwise logistic regression analysis was used, with the latent class with the lowest amount of substance misuse and criminal offending as reference. For selecting variables for the regression-model, ANOVA was used (chi-square for categorical variables).

Results

Could you give an indication of when a score was considered “high” vs “low? (e.g. The second largest class that emerged was named “Alcohol only” (22.21%, n=849, males=420 females=426 missing=3) with the highest alcohol use, but lower scores on everything else.” “Lower” than what? The score of other classes? The score compared to alcohol use? “Moderately” is also used as a term, a definition of this would also be useful to the reader. To be more clear on what is compared to what would aid the reader.

I don’t quite understand the following: “In general, the risk factor profiles of “Alcohol only”, “Polydrug use and crime” and “High crime” differed from “Low/abstainers”, with less favorable scores,…” What does “less favorable” mean?

This formulation: “For belonging to the “Alcohol only” class, higher *parental supervision* and *peer problems* were associated with decreased probability..” is a bit surprising, does this mean that higher degree of peer problems was associated with decreased probability of belonging to the Alcohol only class? So that to only use alcohol seems to be related to less peer problems? (this is related to a comment of mine further below)

Discussion

Maybe not call the classes “interesting” (second paragraph), perhaps define why they’re interesting instead, such as “presenting troubles” etc might be more suited to the aim.

Third paragraph, what “very” denotes here is unclear. Could low/moderate/high/very high be defined somewhere? Also, in this paragraph, it would be suitable with a Swedish reference to drinking/drug habits for comparison also since adolescents in USA and Sweden (especially younger teenagers) seem to differ (e.g. prevalence of cannabis use compared to alcohol use).

The following reference to these results is, at least as it is formulated now, somewhat unsuitable as a reference. “For instance, 68% of violent crimes in Sweden can be attributed to 1% of the population [31]. This corresponds to the high crime class that was identified in this study – a very small group that committed the highest amount of crime.” This study refers primarily to adult persons high in violent crime recidivism, a group not particularly generalizable to late teens due to the considerable prevalence of teenagers who quit their norm-breaking behavior when entering adulthood. The reason for being careful with such generalization in my opinion is to avoid stigma, we don’t know how this latent class develop regarding violent crime in their adult life.

Also the last sentence in this paragraph is not complete: “This class also engaged in co-occurring behaviors (…)” Which?

Check the following sentence: “For example, the “High crime” class had considerable higher mean scores of sensation seeking behavior than the “Low/abstainers” class and the highest mean score of callous unemotional traits, but neither of these risk factors remained significant.” What does “remain” denote here?

Is this opinion backed by either other studies or something in the present data?: “Nevertheless, the risk factors that were significant in this study for the “High crime” and “Polydrug use and crime” classes, might be particularly salient.” If such a statement is made, it could be backed by something from the present data (e.g. a Bayes factor value etc) or clarified that this statement refers to low *n/limited power* and that future studies probably need to use larger samples. Or that future studies should use another approach due to low prevalences in the samples, e.g. a Bayesian approach/odds ratio instead, to be more able to draw conclusions from their data.

I think this is an interesting aspect that can be considered more in-depth: “Considering peer problems, this measures social isolation and expulsion from friend groups of the same age. Peer influence is a strong risk factors for alcohol consumption [12, 13, 35], thus, it is likely that less socializing with others decreased the probability.”. I wonder if belonging to this class is necessarily a problem/bad for the adolescent. In particular if you phrase it “less socializing with others decreased the probability”, which makes less socializing sound like a good thing for the adolescent. Perhaps this is a possibility to discuss the “adolescence-limited” norm-breaking behavior research in light of your results. To break norms together with friends during adolescence, is it always a bad/risky thing for the development? If the alternative may be “social isolation”? I think this requires some more discussion. Perhaps discussing the probability of subgroups within the “Alcohol only”-class, perhaps there is one with destructive use and one with “pro-social” use?

The Discussion could benefit from another check regarding level of abstraction, and to move away from repeating findings (and interpreting them in light of previous research/theories instead). The following section is an example of this: “For instance, the personality trait sensation seeking behavior did increase the probability of belonging to “Alcohol only” and “Polydrug use and crime”, but with considerable higher OR for the latter class Though “polydrug use and crime” showed much higher degree of this. Also, higher parental supervision decreased the probability for both, but “polydrug use and crime” showed lower levels. Similarly, conduct problems increased the probability for “High crime” and “Alcohol only”, but with considerable higher OR for “High crime”. Although, some associations failed to reach significance, “High crime” and “Polydrug use and crime” showed higher levels of hyperactivity than “Alcohol only”, “Polydrug use and crime” also had higher levels of conduct problems than “Alcohol only”, Whereas “High crime” also had higher degree of sensation seeking behavior than “Alcohol only”. In order to examine if risk factor severity contributes to the more maladapted classes, a larger sample is required.”

Strengths and limitation

I don’t quite understand this sentence: “Second, the use of two time-waves of data enables more information on the risk factors implicated in the development of substance use and criminal offending than if the study had been cross-sectional.” Why is this a strength if there is no reference to timing/precedence of risk factors in relation to the latent classes? (which there isn’t in the Discussion as far as I understand). If the “cost” is 1500 responders less than if all data had been gathered at the same time, it is difficult to see this as a strength, especially in light of the power issues discussed.

Also for the reader to understand this as a nationally representative sample and understand the impact of non-responders (both regarding participating schools and follow-up), this would need some additional information on the non-responders. How much more “well-functioning” can we expect the responders to be?

Regarding this sentence: “Also, there is no information beyond this included in this study, which may be relevant for understanding of further development.”, it would help the reader if this information (that could have been useful) was exemplified.

I would be a bit careful with using the word “delopment of” in the following sentence: “Third, factors in adolescence such as less parental supervision, support, peer problems, higher conduct problems, hyperactivity, sensation seeking behavior, distrust in society and truancy are associated with the **development of** and belonging to a class with substance use and/or criminal offending.” since, as I understand it, the temporal sequence of risk factors and class adherence is not certain in the present study. I would just focus on “belonging to”.
