# Supplementary material for: Latent classes of substance use and delinquency in a Swedish national sample of adolescents and associated risk factors
Source: PLoS One. 2025 May 2;20(5):e0322515. doi: 10.1371/journal.pone.0322515 (PMC12047767; doi:10.1371/journal.pone.0322515)
Supplement: Attachment — Submitted filename: Respone to reviewers.docx [file pone.0322515.s004.docx]

Journal Requirements:

When submitting your revision, we need you to address these additional requirements.

1. Please ensure that your manuscript meets PLOS ONE's style requirements, including those for file naming. The PLOS ONE style templates can be found at

https://journals.plos.org/plosone/s/file?id=wjVg/PLOSOne_formatting_sample_main_body.pdf and 
https://journals.plos.org/plosone/s/file?id=ba62/PLOSOne_formatting_sample_title_authors_affiliations.pdf

2. You indicated that you had ethical approval for your study. In your Methods section, please ensure you have also stated whether you obtained consent from parents or guardians of the minors included in the study or whether the research ethics committee or IRB specifically waived the need for their consent.

*Authors’ response: The legal age for consent to participate in research in Sweden is 15, thus consent was not acquired from neither parents or guardians. The clarification “*Consent was only collected from the participants themselves and not from parents or legal guardians. According to the Swedish Act (2003:460) concerning the ethical review of research involving humans, individuals by the age of 15 are legally allowed to consent to participate in research without informing parents or legal guardians [40].” *Has been added to the procedure section in the methods part of the manuscript as well as a reference.*

3. We note that you have indicated that there are restrictions to data sharing for this study. For studies involving human research participant data or other sensitive data, we encourage authors to share de-identified or anonymized data. However, when data cannot be publicly shared for ethical reasons, we allow authors to make their data sets available upon request. For information on unacceptable data access restrictions, please see http://journals.plos.org/plosone/s/data-availability#loc-unacceptable-data-access-restrictions.

Before we proceed with your manuscript, please address the following prompts:

a) If there are ethical or legal restrictions on sharing a de-identified data set, please explain them in detail (e.g., data contain potentially identifying or sensitive patient information, data are owned by a third-party organization, etc.) and who has imposed them (e.g., a Research Ethics Committee or Institutional Review Board, etc.). Please also provide contact information for a data access committee, ethics committee, or other institutional body to which data requests may be sent.

b) If there are no restrictions, please upload the minimal anonymized data set necessary to replicate your study findings to a stable, public repository and provide us with the relevant URLs, DOIs, or accession numbers. Please see http://www.bmj.com/content/340/bmj.c181.long for guidelines on how to de-identify and prepare clinical data for publication. For a list of recommended repositories, please see https://journals.plos.org/plosone/s/recommended-repositories. You also have the option of uploading the data as Supporting Information files, but we would recommend depositing data directly to a data repository if possible.

Please update your Data Availability statement in the submission form accordingly.
 
4. In this instance it seems there may be acceptable restrictions in place that prevent the public sharing of your minimal data. However, in line with our goal of ensuring long-term data availability to all interested researchers, PLOS’ Data Policy states that authors cannot be the sole named individuals responsible for ensuring data access (http://journals.plos.org/plosone/s/data-availability#loc-acceptable-data-sharing-methods).

Data requests to a non-author institutional point of contact, such as a data access or ethics committee, helps guarantee long term stability and availability of data. Providing interested researchers with a durable point of contact ensures data will be accessible even if an author changes email addresses, institutions, or becomes unavailable to answer requests.

Before we proceed with your manuscript, please also provide non-author contact information (phone/email/hyperlink) for a data access committee, ethics committee, or other institutional body to which data requests may be sent. If no institutional body is available to respond to requests for your minimal data, please consider if there any institutional representatives who did not collaborate in the study, and are not listed as authors on the manuscript, who would be able to hold the data and respond to external requests for data access? If so, please provide their contact information (i.e., email address). Please also provide details on how you will ensure persistent or long-term data storage and availability.

*Authors’ response: We understand your open data availability policy. However, we don’t own the data, it is Karolinska Institutet that owns the data. Thus, in order to get assess to data, Karolinska Institutet needs to be contacted (*[*registrator@ki.se*](mailto:registrator@ki.se)*). Further, the survey includes sensitive information on individuals according to the Swedish Personal Data Act. Consequently, an assessment of potential secrecy needs to be conducted by Swedish law. Furthermore, criteria for confidentiality have also been assured when applying for ethical vetting from the Swedish Ethical Review Authority. Given that we can not assure that individuals that will get assess to the data fulfils the criteria for confidentiality, individuals that wants to use the data need a separate ethical vetting from the Swedish Ethical Review Authority before data from Futura01 can be obtained. These considerations have been summarized into the text below.*

“The data for this study are managed by Karolinska Institutet. The Futura01 survey include sensitive information on individuals, as defined by the Swedish Personal Data Act (Personuppgiftslagen, PUL, SFS 1998:204; § 13). Restrictions apply to the availability of these data, which were used under license for this study. Data are available from [registrator@ki.se](mailto:registrator@ki.se) given approval from the Swedish Ethical Review Authority (registrator@etikprovning.se). Access to the data for the purpose of this study was based on membership of a research team with ethical approval for analyses of data from Futura01. Researchers who qualify by getting ethical approval from Swedish authorities can request access to data from the data holder. Data is stored according to the Archives Act (1990:782) and the regulations and general recommendations of the Swedish National Archives concerning the disposal of documents in research activities conducted by government agencies (RA-FS 1999:1).”

5. Please include a caption for figure 1.

*Authors’ response: A caption has been added.*

6. We notice that your supplementary table S1 are included in the manuscript file. Please remove them and upload them with the file type 'Supporting Information'. Please ensure that each Supporting Information file has a legend listed in the manuscript after the references list.

*Authors’ response: The supplementary table has been moved to a separate file.*

Reviewers’ comments

Reviewer 1.

Overall comments. Big sample, suitable methodological choice for the aim, good to focus on SU and criminal offending in synergy, and an important research question (including current relevance). However, there are some issues regarding lack of information on the procedure, definition of groups and interpretation of results that need to be resolved before being able to decide on the study’s contribution to the field (i.e. above all information regarding methodology, see comments below).

Abstract: Regarding Background, clarify whether the aim was to identify risk factors associated *with later in adolescence belonging to specific* latent classes of SU and criminal offending or general risk factors for belonging to any “troubled” class. The four classes obtained represent all possible combinations. Results point to generalized risk factors for any class (including low/abstainers) according to Abstract, or is it only problem classes that is referred to? Which are the “less frequent behaviors” that is referred to in the Abstract? General recommendation regarding the abstract, either choose the most influential factors to present or lift the presentation to a more abstract level (away from specific results), and present it in a more overarching manner. Perhaps also a societal/clinical implication can be mentioned?

*Authors’ response: Thank you for helping us clarify the abstract. Our aim is referring to general and not differentiating factors. We have clarified this by stating that we are examining general factors. We have also revised so that we mention that “abstainers” acted as reference and that factors were associated with classes engaging in substance use and/or delinquency. We have also revised the conclusion and moved away from repeating findings to a more abstract level. The conclusion now reads* ***“*Conclusions:** Most people did not engage in substance use or delinquency. When accounting for less frequent behaviors such as normative adolescent drinking and one-time events of crime and drug use, about 3% of the population engaged in co-occurring substance use and delinquency. Several different factors from several domains where related to belonging to a class that used substances and/or engaged in delinquency. There were indications that the most extensive users and offender displayed a wide variety of severe level risk factors, which could have implications for targeted interventions. Though, statistical power was a problem and future research should use larger samples or alternative methods.*“*

How did they know this risk factor preceded the class adherence (i.e. whether class adherence did not precede the risk factor), and not that the class adherence was already there by the first wave? Or have I misunderstood something basic within the design?*“*

*Authors’ response: We agree with the reviewer. We don’t know if class adherence preceded risk factors. Thus, we have revised a sentence in the third paragraph of the Strength and limitations section.* “It is possible that some risk factors may have been present in earlier stages of life which might be relevant from a developmental perspective, but also that latent class characteristics were present before risk factors.”

Regarding the Aim, a bit of specification would make it clearer: “Thus, the aim of the present study was to identify latent classes of substance use and criminal offending in a national representative Swedish sample and to identify developmental risk factors for each latent class.” Was the aim to identify *differentiating* developmental risk factors or *general* developmental risk factors for all “troubled” latent classes? (Again, I wonder if the developmental risk factors were associated with some kind of temporal assumption, that they preceded the latent class association or that they just were found for this specific/these specific classes?)

*Authors’ response: We are referring to general and not differentiating risk factors. We have clarified this by stating that we are examining general risk factors in the aim.*

Material/method

Regarding participants, could percentages be provided for how many responded to the follow-up? What does “missing” entail here (male/female/missing)? The approx. 1500 participants who did not respond to T2, was any kind of non-response analysis conducted (regarding participant characteristics)? Can the final sample’s characteristics be considered to mirror also the non-responders? I have the same question regarding non-responders for the schools included, did the non-responders differ regarding important characteristics (e.g. SES-status of the area, size of school, rural/town etc)? This issue is touched upon briefly in the Limitations-section but should be considered more in the Discussion.

*Authors’ response: We agree with the reviewer that information of non-responders is lacking. Unfortunately, the data we have only include data on those that participated in both T1 and T2. Thus, we couldn’t perform drop-out analyses. Also, limited comparison between the schools that agreed on participating compare to those who did not agree have been conducted. First, we have revised the “missing” to “Missing information about sex”. Second, we have provided the information we have about differences between schools and have added a sentence in the Participants and procedure section*. “As reported elsewhere, the schools that participated did not differ regarding parental education, immigrant background or average grades compared to schools that did not participate [39].*”*

Material:

I have some questions regarding groups/categories used:

1) Was no standardized form regarding drugs used here? E.g. Dudit?

*Authors’ response: No, there was no standardized form used for drug use in this study. However, the question was constructed similar to the European School Survey Project on Alcohol and Other Drugs (ESPAD) in order to enable comparisons. Drug use was measured with an item that read “How many times, during the latest 12 months, have you used any of the following?” followed by a list of the different substances used. We have revised the text about how drug use was measured into* “Illicit drugs were measured via a battery of questions. The item used for different drugs was “How many times, during the latest 12 months, have you used any of the following?”. Drugs that were included were ‘marijuana’, ‘hashish’, ‘cocaine’, ‘spice/similar smoking substances’, ‘amphetamines’, ‘LSD/other hallucinogens’, and ‘other narcotics’. There was seven response alternatives which ranged from ‘0 times’ (score=0) to ‘40+ times’ (score=6).” *to clarify this.*

2) What was the rationale for including “illegally carrying a weapon” into violent crime?

*Authors’ response: We understand this question since we struggled with this ourselves. There was no clear-cut definition in which category to include carrying a weapon into. The rationale was that carrying a weapon felt more appropriate for and similar to violent crime than theft or property crime*

3) what does the various total scores stand for – does it refer to some kind of ordinal scale numbers/categories? If so, what do the response options denote?

*Authors’ response: Thank you for helping us clarifying this. The total score does stand for index variables made from several ordinal scale items. We have added information throughout the measurements section to clarify what the scores represent an example of such revision: “* … *impulsivity*, which was measured by five items from the Brief Barrat Impulsivity Scale (BIS-Brief) [27], with five response alternatives for each item ranging from ‘strongly disagree’ (score=0) to ‘strongly agree’ (score=4) (total score: 0 – 20, example item: “I easily get angry”, α=0.73) … *“.*

4) This is something that I personally is very interested in and I think could make this manuscript particularly informative: divide SU into alcohol; cannabis; stimulants; sedatives. Since sensation-seeking is investigated, there is a good basis in research to see if crime is particularly related to stimulants rather than sedatives among adolescents. Or check for prevalences/percentages of various drug categories within the respective latent classes. This comment of mine is not necessary to do something about per se, but I think it could give this manuscript an edge and inform the field in an important way. But I understand that group comparisons are difficult due to the power issue.

*Authors’ response: We absolutely agree that it would be much more interesting and informative to make this distinction between different types of substances and investigate this. However, as the way illicit drug use was operationalized (described in one of our previous responses) did not allow us to make this distinction. Since sedative drugs (opioids, benzodiazepines, barbiturates etc.) was not specifically inquired and would fall under “other narcotics” we would not be able to make the comparison with stimulants. Also, as you mentioned, the power issue we had would also make this distinction between various substances difficult. We do however wholeheartedly agree that it would be a very interesting topic and hopefully something for future research to investigate. To highlight this, we have added the sentence “*. Due to the low prevalence and how “other drugs” was measured, a further limitation was that it was not possible to make any comparison between different kind of substances (e.g. comparing sedatives to stimulants etc.) and how they co-occur and relate with delinquency.*” to the limitations-section. We have also added “*Future studies might also want to consider comparing different types of substances and how they relate to and co-occur with delinquency.*” into a paragraph about future research in the Strengths and limitations section of the Discussion to emphasize that we wish future studies to investigate this.*

Regarding the comment that “Some have suggested that correlations greater than 0.5 among indicators might create statistical problems with multicollinearity [29]. However, these indicators were deemed theoretically important and in line with the objective of the study and therefore included.”, it would help the reader if the author clearly notes here what kind of interpretation error that this choice could have resulted in within this study.

*Authors’ response: The interpretation is that multicollinearity in latent class analysis might result in spurious classes appearing. The sentence has been revised into “*Some have suggested that correlations greater than 0.5 among indicators might create statistical problems with multicollinearity that can lead to emergence of spurious classes [50].*“ to clarify this.*

Could you explain a little more to the reader/provide a rational in the text regarding the following analytic steps: To see what factors at T1 predicted classes at T2, pairwise logistic regression analysis was used, with the latent class with the lowest amount of substance misuse and criminal offending as reference. For selecting variables for the regression-model, ANOVA was used (chi-square for categorical variables).

*Authors’ response: We agree with the reviewer that this was unclear. The rationale was to remove any insignificant predictor that did not contribute to the probability for a given class-adherence and hence, minimize the risk of statistically overfitting the regression models. We have tried to clarify the description by revising it to “*To see what factors at T1 predicted classes at T2, pairwise logistic regression analysis was used. For selecting variables for the regression-model, ANOVA was used (chi-square or categorical variables) to identify significant differences between latent classes. These steps of the analysis were performed in IBM SPSS 28 [51]. Logistic regression analyses, where factors with the highest p-value were removed stepwise until only significant factors remained, where then conducted. This was to remove insignificant predictors and reduce the number of predictors to minimize risk of potentially overfitting the model. Biological sex was controlled for in every step of the analyses.*”*

Results

Could you give an indication of when a score was considered “high” vs “low? (e.g. The second largest class that emerged was named “Alcohol only” (22.21%, n=849, males=420 females=426 missing=3) with the highest alcohol use, but lower scores on everything else.” “Lower” than what? The score of other classes? The score compared to alcohol use? “Moderately” is also used as a term, a definition of this would also be useful to the reader. To be more clear on what is compared to what would aid the reader.

*Authors’ response: We agree that our use of high vs. low can be confusing. They should be interpreted in comparison with other latent classes. Thus, we have tried to clarify this in the description of the latent classes. For the “Low/abstainers” class we have added a sentence “*This class was also selected as a reference group for the regression analyses and this was the class with the lowest amount of substance use and delinquency of all classes”. *We revised another sentence in the description of the “Alcohol only” class into “*The second largest class that emerged, which was 22.21% of the sample (n=849, Male=420, Female=426, Missing=3), was named “Alcohol only” with the highest alcohol use of all classes, but with lower scores on everything else than other classes except for “Low/abstainers*”.* *We also revised a sentence in the description of the “Polydrug use and crime” class into* “The third largest class identified, which constituted of 2.15% (n=82, Male=52, Female=30) was named “Polydrug use and crime” and scored in comparison to other classes high on *alcohol*, the highest on *cannabis* and *other drugs*, and the second highest on all crime types.*”*

I don’t quite understand the following: “In general, the risk factor profiles of “Alcohol only”, “Polydrug use and crime” and “High crime” differed from “Low/abstainers”, with less favorable scores,…” What does “less favorable” mean?

*Authors’ response: We agree that it can be confusing, thus we try to clarify by including in brackets what we mean “*(i.e, lower scores on protective factors within “Family factors) and higher on risk factors within the other domains).*”*

This formulation: “For belonging to the “Alcohol only” class, higher *parental supervision* and *peer problems* were associated with decreased probability..” is a bit surprising, does this mean that higher degree of peer problems was associated with decreased probability of belonging to the Alcohol only class? So that to only use alcohol seems to be related to less peer problems? (this is related to a comment of mine further below)

*Authors’ response: Your interpretation is correct. Alcohol use was in this study related to less peer problems. For a more in depth answer and discussion regarding this see our response in your related comment.*

Discussion

Maybe not call the classes “interesting” (second paragraph), perhaps define why they’re interesting instead, such as “presenting troubles” etc might be more suited to the aim.

*Authors’ response: We agree that “interesting” might not have been a suitable word. This has been changed into “*… three classes engaging in delinquency and/or substance use …*”.*

Third paragraph, what “very” denotes here is unclear. Could low/moderate/high/very high be defined somewhere? Also, in this paragraph, it would be suitable with a Swedish reference to drinking/drug habits for comparison also since adolescents in USA and Sweden (especially younger teenagers) seem to differ (e.g. prevalence of cannabis use compared to alcohol use).

*Authors’ response: We have revised to avoid the problems with high. The revised sentence is now formulated “*Almost a quarter were classified into the “Alcohol only” class, where members displayed the highest alcohol consumption of all classes.*” We have also replaced the reference on US drinking with a Swedish. “*Among Swedish 18-year-olds it is estimated that almost a quarter has displayed either harmful use, alcohol dependence or sub-threshold for alcohol dependence [22].*”. We also state within the paragraph that we do not at this stage know which will be adolescent limited drinkers and which will go on to persist drinking into adulthood: “*It still not clear, however, which of these adolescent’s drinking is constrained to the adolescent years and whose is continued into adult years.*”*

The following reference to these results is, at least as it is formulated now, somewhat unsuitable as a reference. “For instance, 68% of violent crimes in Sweden can be attributed to 1% of the population [31]. This corresponds to the high crime class that was identified in this study – a very small group that committed the highest amount of crime.” This study refers primarily to adult persons high in violent crime recidivism, a group not particularly generalizable to late teens due to the considerable prevalence of teenagers who quit their norm-breaking behavior when entering adulthood. The reason for being careful with such generalization in my opinion is to avoid stigma, we don’t know how this latent class develop regarding violent crime in their adult life.

*Authors’ response: This is a very valid point. Our intention was not to equate the “High crime” class to this group of violent offenders in Sweden. Our intention was to highlight the established phenomenon that a very small amount of individuals commit a disproportionate amount of crime. We agree that our wording may have implied equating the two groups and we have revised accordingly. We have also considered the point that rule breaking can be considered to be a normative part of adolescence and a behavior that many cease in adult years. We have highlighted that we at this stage do not have information on how the continued delinquency of the members develops and that some possibly can cease their offending while others may retain it. We have revised this part into “*In the general population, the number of individuals who display a pattern of high frequent delinquent behaviors is often smaller [32]. It has also been established that a small group of individuals commit a disproportionate number of crimes in the general population. An example of this phenomenon is that 68% of violent crimes in Sweden can be attributed to 1% of the population [54]. Another example of this is that of the life course persistent offender in Moffit’s taxonomy. A group of offenders that is very small in size, but commit a disproportionately high frequency of criminal offences and also begin their offending in early adolescence and childhood [53]. This can correspond to the “High crime” class that was identified in this study – a very small group that committed the highest amount of crime. Though in this stage it is not clear how the further criminal behavior of this class will develop. In Moffit’s taxonomy the life course persistent offender differs from the adolescent limited offender who ceases their offending in adulthood. Some delinquent behavior during adolescence can be considered normative and the majority of these phase out in the years to follow [20, 53]. It is not possible to discern in this study which members of “High crime” class will cease their delinquency and who will retain it into adulthood. It could be that some of the members of this class will go on to develop a pattern of offending close to that of the life course persistent offender, but this is at this stage speculative.*”*

Also the last sentence in this paragraph is not complete: “This class also engaged in co-occurring behaviors (…)” Which?

*Authors’ response: We have added a clarification in the end of the sentence. “*… Namely consuming alcohol, illicit drugs and committing crimes.”

Check the following sentence: “For example, the “High crime” class had considerable higher mean scores of sensation seeking behavior than the “Low/abstainers” class and the highest mean score of callous unemotional traits, but neither of these risk factors remained significant.” What does “remain” denote here?

*Authors’ response: “Remained” refers to the factors that were still significant after the stepwise removal of the non-significant factors in the analysis. To clarify this, we added the following at the end of the sentence “* … after the process of eliminating non-significant factors stepwise in the analytical approach.*”*

Is this opinion backed by either other studies or something in the present data?: “Nevertheless, the risk factors that were significant in this study for the “High crime” and “Polydrug use and crime” classes, might be particularly salient.” If such a statement is made, it could be backed by something from the present data (e.g. a Bayes factor value etc) or clarified that this statement refers to low *n/limited power* and that future studies probably need to use larger samples. Or that future studies should use another approach due to low prevalences in the samples, e.g. a Bayesian approach/odds ratio instead, to be more able to draw conclusions from their data.

*Authors’ response: Our reasoning here was indeed referring to the lack of statistical power and low prevalence and that despite this apparent problem these factors remained significant. We have revised the statement into “*Nevertheless, the risk factors that were significant in this study for the “High crime” and “Polydrug use and crime” classes, might be particularly salient due to the fact that they remained significant despite the apparent problem with lack of statistical power and low prevalence of drug use and delinquency in the sample.*” to clarify that this refers to statistical power.*

*We have also added a statement at the limitations-section that suggest future studies to use a larger sample or other measuring techniques such as official registers to reduce this problem. The statements we added were “*With the inability to detect significant risk factors due to low prevalence of substance use and delinquency in the sample and limited statistical power future studies should consider larger samples to reduce this problem. Another strategy for future research is to employ official registers such as medical records and crime registries. Since this study was biased towards those who did not engage in substance use and/or delinquency, official records could be possible way for studies with similar design to reach participants who could not be reached otherwise.*”*

I think this is an interesting aspect that can be considered more in-depth: “Considering peer problems, this measures social isolation and expulsion from friend groups of the same age. Peer influence is a strong risk factors for alcohol consumption [12, 13, 35], thus, it is likely that less socializing with others decreased the probability.”. I wonder if belonging to this class is necessarily a problem/bad for the adolescent. In particular if you phrase it “less socializing with others decreased the probability”, which makes less socializing sound like a good thing for the adolescent. Perhaps this is a possibility to discuss the “adolescence-limited” norm-breaking behavior research in light of your results. To break norms together with friends during adolescence, is it always a bad/risky thing for the development? If the alternative may be “social isolation”? I think this requires some more discussion. Perhaps discussing the probability of subgroups within the “Alcohol only”-class, perhaps there is one with destructive use and one with “pro-social” use?

*Authors’ response: We agree that this is an interesting aspect and we have tried to further discuss this more in-depth. Our intention was not to imply that less socializing was good for the individual, but to highlight since alcohol consumption is a social behavior and peer pressure in adolescence is a strong influence and socializing with others who also use alcohol is an established risk factor, and as isolation would entail less exposure to others who consume alcohol in general, hence why this decreased the probability. We have attempted to revise as to reduce any misunderstanding that we imply that isolation is good. Also, we find it interesting, agree with and have brought up the point that it is common for many adolescents to engage in antisocial behavior with their peers during these years, before ceasing in adult years. It is likely that alcohol consumption can be a manifestation of such behavior and that some members of this class likely will decrease and reduce their drinking habits after adolescence. We have added to and revised this text into “*Considering peer problems, this measures social isolation and expulsion from friend groups of the same age. Peer influence is a strong risk factor for alcohol use and socializing with others who use alcohol increase the risk of consumption [17, 18, 23]. Thus, it is likely that less socializing with others decreased the probability. This does not necessarily mean that socializing with peers is something maladaptive. Although high alcohol consumption is associated with a number of health issues [1], not all adolescent drinkers retain their consumption into adulthood [55]. For some, temporary rule-breaking behavior along with peers can be considered a normative part of adolescence [20, 53]. It seems that this pattern also can apply to alcohol use and after adolescence when peer influence is not as salient, the alcohol consumption will decrease among some members of the “Alcohol only” class.*”.*

The Discussion could benefit from another check regarding level of abstraction, and to move away from repeating findings (and interpreting them in light of previous research/theories instead). The following section is an example of this: “For instance, the personality trait sensation seeking behavior did increase the probability of belonging to “Alcohol only” and “Polydrug use and crime”, but with considerable higher OR for the latter class Though “polydrug use and crime” showed much higher degree of this. Also, higher parental supervision decreased the probability for both, but “polydrug use and crime” showed lower levels. Similarly, conduct problems increased the probability for “High crime” and “Alcohol only”, but with considerable higher OR for “High crime”. Although, some associations failed to reach significance, “High crime” and “Polydrug use and crime” showed higher levels of hyperactivity than “Alcohol only”, “Polydrug use and crime” also had higher levels of conduct problems than “Alcohol only”, Whereas “High crime” also had higher degree of sensation seeking behavior than “Alcohol only”. In order to examine if risk factor severity contributes to the more maladapted classes, a larger sample is required.”

*Authors’ response: We have worked through the discussion in attempt to broaden and deepen it and move away from repeating findings. The paragraph you have brought up as an example has been revised into “*Perhaps, the key to understand the “Polydrug use and crime” and “High crime” classes is similarities in risk profiles when it comes to severity. “High crime” and “Polydrug use and crime” members showed higher means and appears more maladjusted than “Alcohol only”. For instance, the factors that predicted both “Alcohol only”, “Polydrug use and crime” and/or “High crime” (parental supervision, sensation seeking behavior and conduct problems) had stronger association (weaker for parental supervision) to the latter groups. The latter groups also had higher levels of these risks than “Alcohol only”. “Polydrug use and crime” and “High crime” also consistently had higher level of risk among all factors that reached significance for “Alcohol only”, but not for the aforementioned classes. It has been emphasized previously that the most serious antisocial individual, such as the life course persistent offender in Moffit’s taxonomy, display both problematic individual and familial factors [53]. Factors that both “Polydrug use and crime” and “High crime” displayed high levels of. Risk factors also have a cumulative effect, where more factors present leads to a higher risk of an outcome [56]. The “Polydrug use and crime” and “High crime” classes could possibly also have a wider range of different risk factors present, leading to problematic behaviors. In order to examine if risk factor severity and accumulation contributes to these more maladapted classes, a larger sample is required.*”. In addition to the comments which you have brought up regarding the discussion and our responses we have also added “*This class also did not engage in any extensive delinquency despite having the highest alcohol consumption. This is notable since alcohol has been previously implied to be a contributing factor to criminal behavior, especially aggression and violence [14]. However, some research imply that alcohol use does not cause this behavior in itself, but rather directs it onto things such as provoking or inhibitory cues [52]. Also, it has been implied that the most frequent and persistent criminal offenders are driven by different familial and pathological factors early on in life, such as family problems, mental health issues and problematic personality [53]. It may be that there are other factors which are more driving in delinquency than only alcohol consumption., This could be possible, since judging by the mean scores of risk factors among the classes that engaged in substance use and/or delinquency, the “Alcohol only” class seemed to be the most well-adjusted. Though, it should be noted that this is not something that has been tested in this study and therefore remains speculative.*”. and “*Further, a “Polydrug use and crime” class was identified with co-occurrence of multiple substances, both alcohol, cannabis and other drugs, and acts of delinquency. This class had high alcohol consumption, though they were still consuming less than “Alcohol only”. This class also had the highest illicit drug use compared to the other classes, particularly cannabis. This class was also more likely to commit crime than both the “Low/Abstainers” class and the “Alcohol only” class. Though this was difference was not as large as between the “High crime” class and the other classes.*” when talking about “alcohol only” and “polydrug use and crime” respectively. We have also added a section on implications “*There are some implications with these results. Firstly, since there are indications that extensive substance use and delinquency seems to be limited to small part of the general population and most are not in need of any interventional actions. Interventions may be located and focused to where they are most needed instead. Though, this should be done with caution as targeted interventions may run the risk of labeling and stigmatizing individuals. Secondly, this study and research before indicate that the risk factor profile of the most severe substance users and delinquent offenders is multifactorial. The most extensive users and offenders have a high level and wide range of several different risk factors. It may be that interventions also need to be multifactorial and cover several different problem areas, such as improving mental health, family conditions, peer relations and school environment etc., in order to diminish and prevent further substance use and delinquent behaviors in the future.*” and we have rewritten the conclusion “*This study sought to identify different latent classes of substance misuse and delinquency in adolescence, and developmental risk factors associated with them. The vast majority of the participants do not engage in any extensive substance misuse or delinquency. The most common form of substance use was alcohol, likely adolescent binge drinking, and the most extensive substance use and delinquency was attributed about 3% of the sample. Substance users and delinquent offenders also show a multifactorial risk profile where several factors, such as familial problems, mental health mental health issues, personality traits, peer factors and other factors are related to belonging to a class with substance use and/or committing delinquency. This has implications that interventions might need to cover several factors as well. A relatively short time window has been investigated and future research should investigate longer time frames to establish prevalence of which limit their consumption/delinquency to adolescence and which persist into adult years. Statistical power was also a problem in this study and future research should use larger samples or other methods in an attempt to improve this.*”*

Strengths and limitation

I don’t quite understand this sentence: “Second, the use of two time-waves of data enables more information on the risk factors implicated in the development of substance use and criminal offending than if the study had been cross-sectional.” Why is this a strength if there is no reference to timing/precedence of risk factors in relation to the latent classes? (which there isn’t in the Discussion as far as I understand). If the “cost” is 1500 responders less than if all data had been gathered at the same time, it is difficult to see this as a strength, especially in light of the power issues discussed.

Also for the reader to understand this as a nationally representative sample and understand the impact of non-responders (both regarding participating schools and follow-up), this would need some additional information on the non-responders. How much more “well-functioning” can we expect the responders to be?

Regarding this sentence: “Also, there is no information beyond this included in this study, which may be relevant for understanding of further development.”, it would help the reader if this information (that could have been useful) was exemplified.

*Authors’ response: We totally understand your point about the two time-waves data and have excluded this as a strength.*

*Regarding the last sentence of the Strength and limitations section, we agree that this sentence is confusing. We have now revised it into “*Also, there is no information beyond the age of 17/18, which may be relevant for understanding of further development.”

I would be a bit careful with using the word “delopment of” in the following sentence: “Third, factors in adolescence such as less parental supervision, support, peer problems, higher conduct problems, hyperactivity, sensation seeking behavior, distrust in society and truancy are associated with the **development of** and belonging to a class with substance use and/or criminal offending.” since, as I understand it, the temporal sequence of risk factors and class adherence is not certain in the present study. I would just focus on “belonging to”.

*Authors’ response: “Development of” has been removed and the focus in now solely on belonging to. The sentence has been revised into “* … are related with belonging to a class with substance use and/or committing delinquency.*”.*

Reviewer 2.

Reviewer #2: The current study has potential but suffers from some important problems, which fortunately can be rectified.
1. The engagement of the literature is weak which is problematic because there are latent class papers of the crime/substance use overlap. Several of these are listed below and were based on the NESARC, NSDUH, and other epidemiological data.
a. Vaughn, M. G., Salas-Wright, C. P., Delisi, M., & Piquero, A. R. (2014). Health associations of drug-involved and criminal-justice-involved adults in the United States. Criminal Justice and Behavior, 41(3), 318-336.
b. DeLisi, M., Vaughn, M. G., Salas-Wright, C. P., & Jennings, W. G. (2015). Drugged and dangerous: Prevalence and variants of substance use comorbidity among seriously violent offenders in the United States. Journal of Drug Issues, 45(3), 232-248.
c. Fearn, N. E., Vaughn, M. G., Nelson, E. J., Salas-Wright, C. P., DeLisi, M., & Qian, Z. (2016). Trends and correlates of substance use disorders among probationers and parolees in the United States 2002–2014. Drug and alcohol dependence, 167, 128-139.
d. Kendler, K. S., Ohlsson, H., Sundquist, K., & Sundquist, J. (2013). A latent class analysis of drug abuse in a national Swedish sample. Psychological Medicine, 43(10), 2169-2178.

*Authors’ response: Thank you for providing literature so we can reinforce the introduction. We have attempted to improve the introduction by including more literature and information on the co-occurrence of substance use and crime. We have added literature in the paragraph describing previous studies who analyzed classes of co-occurring behaviors.*

*Firstly, We have revised the seventh paragraph where we after the two introductory sentences have added literature and revised the text into: “*For the development of co-occurring substance use and delinquency, classes of both substance use and delinquency are essential. However, only a few studies have taken this approach. One study on American violent offenders aged 12-25, found that almost 40 % had indications of different types of substance use disorders, a larger group of alcohol and marijuana use disorders and a smaller for polysubstance use disorder [35]. Another study examined different classes of Swedes born between 1950-1993 registered for drug use by using the Swedish medical and crime registers. Two classes who engaged in criminal behavior, one larger with low frequency of drug use and one smaller with high frequency of drug use, and two classes who only engaged in drug use, one larger with lower frequency and one smaller with higher frequency, were identified [36]. Other studies have also identified classes of substance use and delinquency among risk populations such as inhalant users [37] or youths involved in diversion programs [17]. Since these studies are examining risk populations, it is not clear how these results translate to general population of adolescents. One study identified three latent classes among 548 adolescents aged 12-17 years recruited from two schools in Australia, where the most adapted class engaged in underage alcohol use only, whereas two other classes that were more heavily engaged in delinquency also used illicit drugs [38]. This study used dichotomized items (yes/no), neglecting frequency of behaviors. Consequently, knowledge of subgroups that engage in both substance use and delinquency is scarce, particularly as derived from large national representative samples.*”*

*Secondly, we have also in the opening paragraphs added information on the global prevalence of substance use disorders, interpersonal violence and victimization of crime in Sweden. We also added more information in the opening on the health consequences of individuals who engage in drug use and criminal behavior. This paragraph now reads “*Substance use and delinquency constitute a burden for public health, since substance use is associated with premature death, infectious diseases and poorer mental health [1, 2], while being a victim of crime is associated with premature death and negative health conditions [3, 4]. Individuals who are involved with both drug use and criminal behavior are also at a risk of developing health issues related to a long-term antisocial lifestyle such as hepatitis, pancreatitis, and sexually transmitted diseases [5]. Both behaviors are common. It is estimated that there are 1320.8 alcohol use disorders per 100 000 people and between 52.1 to 289.7 various drug use disorders per 100 000 people globally [1]. Also, interpersonal violence is the fifth leading cause globally for injuries [6]. It has also been reported that a fifth of the population in Sweden has been the victim of a crime [7].*“*

*Thirdly, in the following paragraph we also added more information on the prevalence of co-occurring behaviors. This paragraph now reads “*Substance use and crime tend to co-occur in an individual. It is estimated that a quarter of incarcerated prisoners meet the criteria for alcohol use disorder and close to a third of prisoners meet the criteria for some type of drug use disorder [8]. Also, the prevalence of substance use disorders among individuals released on parole or on probationary sentencing are estimated to up to nine times higher than in individuals who have not had any contact with the criminal justice system [9].*“*

*We have also added more information on why the focus on co-occurrence is important by highlighting how co-occurring behaviors are more detrimental than single behaviors in the following paragraph. The sentences “*Drug use can also contribute to an escalation of criminal activity and frequency of crime is higher during periods when use is also high. Substance use can also prolong the trajectory of criminal behaviors. Substance users seem to take a longer time to phase out of criminal activities than non-users [11, 12, 13].*“ have been revised and added to the paragraph.*

*Finally we have added information on the prevalence of substance use and delinquency among Swedish adolescents and common risk factors for the development of these behaviors in the paragraph regarding adolescents in the introduction. The sentences “*It is estimated that about 13% of Swedish 18-year-olds fulfil the criteria for substance use disorder and a considerable part are at risk of fulfilling criteria for such a disorder [22]. Also, it is estimated among Swedish adolescents that by age 19 almost a fifth of males and 5% of females have been registered for a criminal act [20]. Thus, it seems that adolescence is a period where one is susceptible to the risk of developing substance use and delinquent behaviors. Common risk factors in adolescence are familial problems and negative parenting styles [23, 24, 25], antisocial peers, and peer pressure [17, 18, 23, 25], mental health problems such as hyperactivity, conduct problems and having symptoms of conduct disorder [25, 26, 27, 28, 29] and problematic personality traits like impulsivity, sensation seeking and psychopathic personality traits [27, 28, 29, 30].*“ have been added to the paragraph.*

2. The discussion largely reiterates the findings and again the engagement of the literature is weak. It seems the findings have implications for Moffitt’s developmental taxonomy.
a. Moffitt, T. E. (1993). Adolescence-limited and life-course-persistent antisocial behavior: a developmental taxonomy. Psychological Review, 100, 674-701.
b. Moffitt, T. E. (2018). Male antisocial behaviour in adolescence and beyond. Nature human behaviour, 2(3), 177-186.

*We have worked through the discussion in an attempt to broaden and deepen it. We have taken up on your comment and have attempted to integrate Moffit’s taxonomy throughout the discussion. In particular we emphasize the difference in trajectories among the adolescence limited and life course persistent offenders, and draw likeness to substance consumption as well. We also, emphasize that the most prolific offenders (the life course persistent) show a risk profile of several different environmental and pathological factors early on in life and that our result seem to indicate this as well. Examples of where we have integrated this into the manuscript are “*Moreover, a “High crime” class was identified who scored the highest on all crime types, considerably higher for every crime type compared to “Polydrug use and crime” who was the second highest for crime among the classes. This class also engaged in co-occurring behaviors, namely consuming alcohol, illicit drugs and committing crimes. The alcohol consumption of the “High crime” class was also considerably higher than for the “Low/abstainers” class. Also, this class had the second highest illicit drug use. In the general population, the number of individuals who display a pattern of high frequent delinquent behaviors is often smaller [32]. It has also been established that a small group of individuals commit a disproportionate number of crimes in the general population. An example of this phenomenon is that 68% of violent crimes in Sweden can be attributed to 1% of the population [54]. Another example of this is that of the life course persistent offender in Moffit’s taxonomy. A group of offenders that is very small in size, but commit a disproportionately high frequency of criminal offences and also begin their offending in early adolescence and childhood [53]. This can correspond to the “High crime” class that was identified in this study – a very small group that committed the highest amount of crime. Though in this stage it is not clear how the further criminal behavior of this class will develop. In Moffit’s taxonomy the life course persistent offender differs from the adolescent limited offender who ceases their offending in adulthood. Some delinquent behavior during adolescence is not uncommon and the majority of these phase out in the years to follow [20, 53]. It is not possible to discern in this study which members of “High crime” class will cease their delinquency and who will retain it into adulthood. It could be that some of the members of this class will go on to develop a pattern of offending close to that of the life course persistent offender, but this is at this stage speculative.*”, “*This class also did not engage in any extensive delinquency despite having the highest alcohol consumption. This is notable since alcohol has been previously implied to be a contributing factor to criminal behavior, especially aggression and violence [14]. However, it may be that there are other factors which are more driving in delinquency than only alcohol consumption. Some research imply that alcohol use does not cause this behavior in itself, but rather directs it onto things such as provoking or inhibitory cues [52]. It has also been implied that the most frequent and persistent criminal offenders are driven by different environmental and pathological factors early on in life, such as family problems, mental health issues and problematic personality [53]. This could be possible, since judging by the mean scores of risk factors among the classes that engaged in substance use and/or delinquency, the “Alcohol only” class seemed to be the most well-adjusted. Though, it should be noted that this is not something that has been tested in this study and therefore remains speculative.*”, “*Although high alcohol consumption is associated with a number of health issues [1], not all adolescent drinkers retain their consumption into adulthood [55]. For some, temporary rule-breaking behavior along with peers can be considered a normative part of adolescence [20, 53]. It seems that this pattern also can apply to alcohol use and after adolescence, when peer influence is not as salient, the alcohol consumption might decrease among some members of the “Alcohol only” class.*” and “*It has been emphasized previously that the most serious antisocial individual, such as the life course persistent offender in Moffit’s taxonomy, display both problematic individual and familial factors [53]. Factors that both “Polydrug use and crime” and “High crime” displayed high levels of. Risk factors also have a cumulative effect, where more factors present leads to a higher risk of an outcome [56]. The “Polydrug use and crime” and “High crime” classes could possibly also have a wider range of different risk factors present, leading to problematic behaviors. In order to examine if risk factor severity and accumulation contributes to these more maladapted classes, a larger sample is required.*”*. *In addition to this we have also added a section for implications “*There are some implications with these results. Firstly, since there are implications that extensive substance use and delinquency seems to be limited to small part of the general population and most are not in need of any interventional actions. Interventions may be located and focused to where they are most needed instead. Though, this should be done with caution as targeted interventions may run the risk of labeling and stigmatizing individuals. Secondly, this study and research before indicate that the risk factor profile of the most severe substance users and delinquent offenders is multifactorial. The most extensive users and offenders have a high level and wide range of several different risk factors. It may be that interventions also need to be multifactorial and cover several different problem areas, such as improving mental health, family conditions, peer relations and school environment etc., in order to diminish and prevent further substance use and delinquent behaviors in the future.*”, suggestions for future research “*With the inability to detect significant risk factors due to low prevalence of substance use and delinquency in the sample and limited statistical power, future studies should consider larger samples to reduce this problem. Another strategy for future research is to employ official registers such as medical records and crime registries. Since this study was biased towards those who did not engage in substance use and/or delinquency, official records could be possible way for studies with similar design to reach participants who could not be reached otherwise. Future studies might also want to consider comparing different types of substances and how the relate to and co-occur with delinquency. Also, longer time windows should be investigated which enables to examine developmental trajectories and differences between those whose substance use and delinquency is limited to adolescent years and those persist into later years.*” and we have rewritten the conclusion “*This study sought to identify different latent classes of substance misuse and delinquency in adolescence, and developmental risk factors associated with them. The vast majority of the participants do not engage in any extensive substance misuse or delinquency. The most common form of substance use was alcohol, likely adolescent binge drinking, and the most extensive substance use and delinquency was attributed about 3% of the sample. Substance users and delinquent offenders also show a multifactorial risk profile where several factors, such as familial problems, mental health mental health issues, personality traits, peer factors and other factors are related to belonging to a class with substance use and/or committing delinquency. This has implications that interventions might need to cover several factors as well. A relatively short time window has been investigated and future research should investigate longer time frames to establish prevalence of which limit their consumption/delinquency to adolescence and which persist into adult years. Statistical power was also a problem in this study and future research should use larger samples or other methods in an attempt to improve this.*”*

3. As the author notes, the cell sizes for polydrug use and crime and high crime latent classes are very small. Did you consider combining them? Perhaps forcing a 3-class solution makes more sense given that nearly 75% are abstainers and another 22% are normative alcohol only users.

*Authors’ response: This is something that we have given thought to. We did expect the most “extreme” class (High crime) to be of very small size since it is known in criminological literature that a very small group of individuals commit a disproportionate amount of crime, which we touch briefly upon in the discussion. Also, we had in mind the life-course persistent offender of Moffit’s taxonomy which you mentioned in the previous comment which is similar in size to the “high crime”-class. We also expected a smaller class of polysubstance use, we were just not sure how large. Although from purely a statistical standpoint, combining them would make sense for power. However, from a theoretical perspective we felt that they were behaviorally different, as one class was more focused on committing crime and one was more focused on using drugs. Also, since they corresponded in size to what has previously been observed in the literature, we felt that it was justified to keep them separate.*

Reviewer 3.

A large nationwide study investigated the co-occurrence of self-reported substance use and criminal offending at ages 17/18, as well as whether a range of risk factors, including family, personality, mental health, peers, and others, measured at ages 15/16, predicted these co-occurrences. The findings revealed that it was possible to distinguish four classes of individuals based on their substance use (or non-use) and criminal behavior. Of these, only two classes were characterized by both substance use and criminal behavior, although they were very small. Specific factors appeared to be significant predictors of class membership. Overall, the topic is relevant to the field of study, although I have raised several points that should be addressed before this study can be considered for publication.

In general

Consider using the terms "delinquency" or "delinquent behavior" instead of "criminal behavior," as these terms are more appropriate when discussing adolescence.

*Authors’ response: We agree with this observation and the term “criminal offending” has throughout the manuscript been changed into either “delinquency” or “delinquent behaviors”*

Abstract

It would be useful to include the gender distribution in parentheses.

*Authors’ response: This has been added.*

A large portion of the conclusion merely repeats the results. I recommend rewriting the conclusion section to focus on the implications of these findings, rather than restating the results.

*Authors’ response: We have revised the conclusion in an attempt to move away from repeating the results and focus on a better conclusion. The conclusion has been revised into “***Conclusions:** Most people did not engage in substance use or delinquency. When accounting for less frequent behaviors such as normative adolescent drinking and one-time events of crime and drug use, about 3% of the population engaged in co-occurring substance use and delinquency. Several different factors from several domains where related to belonging to a class that used substances and/or engaged in delinquency. There were indications that the most extensive users and offender displayed a wide variety of severe level risk factors, which could have implications for targeted interventions. Though, statistical power was a problem and future research should use larger samples or alternative methods.*”*

Introduction

The first paragraph of the introduction could be strengthened by including more information on the global prevalence of both substance use and criminal behavior among adolescents, with a particular focus on Sweden, as this was the target population in your study. Including this background in the introduction can help better frame the outcomes of your study and underscore the relevance of your findings within both a global and national context.
In addition, within the same paragraph, when emphasizing the importance of your research, it would be beneficial to focus more specifically on the significance of studying the co-occurrence of substance use and delinquency in adolescence.

*Authors’ response: We agree with the reviewer and have added more information on the prevalence of substance use and violence globally to the first paragraph and also crime victimization in Sweden. We have also added more on the health outcomes in individuals who engage in substance use and crime to this paragraph. We have added the sentences “*Individuals who are involved with both drug use and criminal behavior are also at a risk of developing health issues related to a long-term antisocial lifestyle such as hepatitis, pancreatis, and sexually transmitted diseases [5]. Both behaviors are common. It is estimated that there are 1320.8 alcohol use disorders per 100 000 people and between 52.1 to 289.7 various drug use disorders per 100 000 people globally [1]. Also, interpersonal violence is the fifth leading cause globally for injuries [6]. It has also been reported that a fifth of the population in Sweden has been the victim of a crime [7].*“ to this paragraph. We have also added information on the prevalence among Swedish adolescents in the paragraph about adolescence in the introduction which we mention in our response in one of your comments about the paragraph about adolescence.*

*In addition to this we have added more information on the prevalence of co-occurring behaviors and information on how co-occurring behaviors might be more detrimental than single behaviors. The sentences ”*It is estimated that a quarter of incarcerated prisoners meet the criteria for alcohol use disorder and close to a third of prisoners meet the criteria for some type of drug use disorder [8]. Also, the prevalence of substance use disorders among individuals released on parole or on probationary sentencing are estimated to up to nine times higher than in individuals who have not had any contact with the criminal justice system [9]*.” and “*Drug use can also contribute to an escalation of criminal activity and frequency of crime is higher during periods when use is also high. Substance can also prolong the trajectory of criminal behaviors. Substance users seem to take a longer time to phase out of criminal activities than non-users [11, 12, 13].*“ to the introduction highlighting the importance of focusing on co-occurring behaviors*

The continuation of the introduction could benefit from a more specific explanation of why adolescents are particularly susceptible to engaging in substance use and criminal behavior, along with a focus on the most common and important risk factors

*Authors’ response: Thank you for asking us to improve this. We now highlight in the introduction that adolescence is the period where substance use and delinquency most often have their onset and peaks in these years and also that risk behaviors are a common and considered to be a normative part of these years. We have also added information on prevalence of substance use and delinquency among Swedish adolescents to this paragraph and examples of common risk factors in adolescence. We have revised the paragraph into “*From a developmental perspective, it seems that adolescence is a key period. This is the life-stage where both substance use and delinquency often have their onset, progress and peak through [19, 20]. It is also common that adolescents tend to engage in multiple types of risk behaviors and this might be considered a normative part of adolescence [21]. It is estimated that about 13% of Swedish 18-year-olds fulfil the criteria for substance use disorder and a considerable part are at risk of fulfilling criteria for such a disorder [22]. Also, it is estimated among Swedish adolescents that by age 19 almost a fifth of males and 5% of females have been registered at least once for a criminal act [20]. Thus, it seems that adolescence is a period where one is susceptible to the risk of developing substance use and delinquent behaviors. Common risk factors in adolescence are familial problems and negative parenting styles [23, 24, 25], antisocial peers, and peer pressure [17, 18, 23, 25], mental health problems such as hyperactivity, conduct problems and having symptoms of conduct disorder [25, 26, 27, 28, 29] and problematic personality traits like impulsivity, sensation seeking and psychopathic personality traits [27, 28, 29, 30]. Though there is knowledge on adolescent risk factors for substance use and delinquency, there is less knowledge about the patterns and mechanisms through which substance use and delinquency tend to combine into co-occurring behaviors in this stage of life. One approach to explore such processes is to find underlying subgroups of patterns of substance use and delinquency and which factors are associated with these patterns.*”*

How were substance use and criminal behavior operationalized in your study?

*Authors’ response: As we mention further down in the methods section. Substance use was operationalized by self-reported alcohol use (measured with AUDIT-C) and illicit drug use (measured by a battery of questions which we explain both in the method section and in our response to a comment below) Criminal behavior was operationalized by self-reported violent crime, property crime and theft which me mention how it was measured in the method section.*

In the section on the present study, it would be useful to specify which particular risk factors you focused on and provide a rationale for why these were selected. For example, if you chose to examine family dynamics, peer influence, or mental health problems, explaining how these factors are linked to both substance use and delinquency in your specific context would strengthen your research justification.

*Authors’ response: We have added examples of risk factors to the paragraph about the period of adolescence which we describe in a response above.*

Methods

Participants and Procedure

Were there any inclusion or exclusion criteria for participation in this study?

*Authors’ response: Apart from being a Swedish adolescent born in 2001 there were no inclusion or exclusion criteria.*

Please provide more information on sample characteristics (e.g., mean age, ethnicity, SES, etc.) either in the text or in a table.

*Authors’ response: All participants were all born in the same year (2001). Thus, the participants had the same age when data was collected. We did not inquire about ethnicity. SES (parental income, parental education) has also not been inquired in the surveys in this study. We have provided the information on the sample that we have.*

Measurements

The section on measures would benefit from additional subheadings to group measurements of the same category under a common heading.

*Authors’ response: Thank you for improving the clarity of the manuscript. The measurements section has now been split into the subsections “outcomes” and “risk factors” and headings have been added.*

Could you provide some examples of how you measured "Other drugs"?

*Authors’ response: We agree that it was not made clear how ‘Other drugs’ was measured. Other drugs was measured in the same way cannabis was, which was by an item that read “How many times, during the latest 12 months, have you used any of the following?” followed by a list of different substances. We have revised the text about drug use measurement into* “Illicit drugs were measured via a battery of questions. The item used for different drugs was “How many times, during the latest 12 months, have you used any of the following?”. Drugs that were included were ‘marijuana’, ‘hashish’, ‘cocaine’, ‘spice/similar smoking substances’, ‘amphetamines’, ‘LSD/other hallucinogens’, and ‘other narcotics’. There were seven response alternatives which ranged from ‘0 times’ (score=0) to ‘40+ times’ (score=6).” *to clarify this. The grouping of substances was made stated in the following sentences “*Illicit drugs were categorized into *cannabis* and *other drugs*. *Cannabis* contained marijuana and hashish (total score=0-12, α=0.81, two items). *Other drugs* included the use of the other narcotics listed (total score: 0-30, α=0.77, five items).*”*

You repeat the sentence "Risk factors were measured at T1" twice. Consider removing it from the first paragraph of the section on measures to avoid redundancy.

*Authors’ response: We agree with this comment and one of these sentences has now been removed*

The reliability of the measure for callous-unemotional traits was poor. It would be useful to include the mean item correlation and indicate whether it falls within the acceptable range (for more detail, see Clark & Watson, 1995).
Clark, L. A., & Watson, D. (1995). Constructing validity: Basic issues in objective scale development. Psychological Assessment, 7(3), 309–319. <https://doi.org/10.1037/1040-3590.7.3.309>

*Authors’ response: Thank you for this suggestion. We have checked this and the mean inter-item correlation for this factor was 0.27 which falls within acceptable range. We have added “*This value was 0.27 and was within acceptable ranges [48].*” after the description of CU-traits to clarify this.*

Analysis

Please specify which software you used to conduct the latent class analysis.

*Authors’ response: The LCA was made in Stata 18. A mention and citation has been added.*

Based on the literature, BIC is generally regarded as the most reliable criterion for determining the optimal number of classes. However, I recommend also considering entropy, classification error, class sizes, and whether the identified classes are meaningful and consistent with theoretical assumptions or prior research.

*Authors’ response: Thank you for reminding us on these important notions. Log-likelihood was also examined, this statistic also supported a 4-class solution. From theory we expected also the classes who used substances and committed crime to be smaller, than non-users/non-offenders, but we had no official cutoff for how small the classes were allowed to be. We have revised the statement into “*The best model fit (number of classes) was determined by Akaike’s information criterion (AIC) and Bayesian information criterion (BIC), where smaller values indicate better model fit [50]. In addition to these, log-likelihood was also examined where higher values indicate a better fit. Also, considering previous literature, smaller classes who engaged in substance use/delinquency were expected. Though, there was no official cutoff for how small classes could be*”. We would have also considered entropy, but our statistical software did not allow us to calculate entropy reliably, therefore we left it out.*

Correlations and other statistical findings should be reported in the Results section, not the Methods section.

*Authors’ response: Thank you for this advice. This has been moved to the results section*

I suggest that the reference group be mentioned in the Results section rather than in the Methods section, as at this point, it is not yet clear whether classes will be identified among the indicators.

*Authors’ response: This has been moved to the results section*

Which software did you use to conduct the ANOVA analysis, and what was the initial number of risk factors considered?

*Authors’ response: IBM SPSS 28 was used. A mention and citation has been added.*

Results

Please remove (AIC=52171.21, BIC=52377.40) from the text, as it is not informative on its own.

*Authors’ response: This has been removed*

Table 1 should include additional relevant information, such as the number of parameters, likelihood ratio or log-likelihood, degrees of freedom, p-value, class error, and the entropy of R2.

*Authors’ response: We have added the log-likelihood to the table as we also used this to judge model-fit. As we mentioned in on of our previous responses, we were not able to calculate entropy reliably and therefore left it out.*

In Table 2, you should also include the Wald test, as well as the Wald test for paired comparisons.

*Authors’ response: We have conducted the Wald test for all indicator variables in the model and added the statistic to the table. The results were significant throughout all indicators.*

In the second paragraph of the Results section, the missing values in parentheses are surprising, given that it was previously mentioned that listwise deletion was employed.

*Authors’ response: The parentheses refer to the size of and gender distribution within the identified classes. The missing values denotes the individuals within the classes that we did not have information on biological sex for (11 for abstainers, and 3 for alcohol only). Since Biological sex was not an estimator variable, listwise deletion would not have an effect on this.*

In addition, to enhance readability, avoid placing all information in parentheses. You can write:
The largest class, which comprised 74.8 % of the sample (n = #, % females), scored the lowest on all outcomes and was therefore labeled the “Low/abstainers” class.

*Authors’ response: We agree that this enhances readability. We have taken this into consideration and revised the text into “*The largest class, which comprised 74.80% of the sample (n=2858, Male=1191, Female=1656, Missing=11), had the lowest scores on all outcomes and was named “Low/abstainers”. This class was also selected as a reference group for the regression analyses as this was the class with the lowest amount of substance use and delinquency among all classes. The second largest class that emerged, which was 22.21% of the sample (n=849, Male=420 Female=426, Missing=3), was named “Alcohol only” with the highest alcohol use, but lower scores on everything else compared to the other classes except for “Low/abstainers”. The third largest class identified, which consisted of 2.15% (n=82, Male=52, Female=30), was named “Polydrug use and crime” and scored in comparison to the other classes high on *alcohol*, the highest on *cannabis* and *other drugs*, and the second highest on all crime types. The smallest identified class, which was 0.84% (n=32, Male=30, Female=2), was named “High crime” which scored the highest on all crime types, the third highest on *alcohol* and the second highest for *cannabis* and *other drugs*.*”*

The third and fourth classes are very small, which raises questions about their meaningfulness. This relates to my earlier comment about providing more background information on the prevalence of substance use and delinquency in Sweden, as well as offering more detail in the Methods section regarding what class size would be considered acceptable.

*Authors’ response: We have provided more information on the prevalence of substance use/delinquency in Sweden in the introduction which we mention in our previous responses. We have also added based on literature what class size we expected in the methods section which we highlight in one of our previous responses.*

Table 3 should include information on the F-test or Chi-Square test. Additionally, the Bonferroni correction should be applied for multiple pairwise comparisons.

*Authors’ response: We have added the F values and the Chi Square values to the table. We have also run the post hoc tests with a Bonferroni correction. This application did not change which differences were significant in post hoc tests and thus did not affect which factors were selected for the logistic regression analyses.*

The last paragraph of the Results section is unclear regarding which factors were excluded. To improve clarity and make it easier to follow, please mention the excluded factors in the text.

*Authors’ response: The excluded factors have now been listed in the text. The sentences “ Parental rules* did not differ significantly from “Low/abstainers” among any of the groups and was excluded from the regression analysis for all classes.*” at the end of the second last paragraph and* *“*The excluded factors after ANOVA tests and regression analyses were; *parental rules*, *emotional problems*, *impulsivity*, *callous unemotional traits* and *bullying others* for all classes; *parental supervision* for “High crime”; *parental support* for “Alcohol only”; *hyperactivity* for “Polydrug use and crime” and “High Crime; *conduct problems* for “Polydrug use and crime; *sensation seeking behavior* for “High crime”; *peer problems* for “Polydrug use and crime” and “High crime”; *truancy* for “Alcohol only” and “High crime”; and *distrust in society* for “Alcohol only” and “Polydrug use and crime”.*” at the last paragraph.*

Discussion

In general, the discussion is somewhat limited and would benefit from a deeper analysis grounded in existing literature and empirical findings related to substance use and delinquency in adolescents.
Emphasizing that three classes were identified at T2 may cause confusion, as the first class was also derived from T2 measures. Clarifying this point would improve the paper's flow. Additionally, I recommend avoiding abbreviations and statistical terms in the discussion to enhance readability and clarity.

*Authors’ response: We have removed emphasizing that three classes were identified at T2 as we agree this causes confusion and does not contribute. We have also removed the use of statistical terms and abbreviations in the discussion. We have also worked through the discussion in an attempt to deepen and broaden it. In addition to your comments for which we respond below, when referring to the level of delinquency and drug consumption we emphasize that this is in comparison between the identified classes. When discussing risk factors, based on a comment from another reviewer we have also added and revised the statements “*Considering peer problems, this measures social isolation and expulsion from friend groups of the same age. Peer influence is a strong risk factor for alcohol use, and socializing with others who use alcohol increases the risk of consumption [17, 18, 23]. Thus, it is likely that less socializing with others decreased the probability. This does not necessarily mean that socializing with peers is something maladaptive. Although high alcohol consumption is associated with a number of health issues [1], not all adolescent drinkers retain their consumption into adulthood [55]. For some, temporary rule-breaking behavior along with peers can be considered a normative part of adolescence [20, 53]. It seems that this pattern also can apply to alcohol use and after adolescence, when peer influence is not as salient, the alcohol consumption might decrease among some members of the “Alcohol only” class.*”,”* Nevertheless, the risk factors that were significant in this study for the “High crime” and “Polydrug use and crime” classes, might be particularly salient due to the fact that they remained significant despite the apparent problem with lack of statistical power and low prevalence of drug use and delinquency in the sample.*” and “*Perhaps, the key to understand the “Polydrug use and crime” and “High crime” classes is similarities in risk profiles when it comes to severity. “High crime” and “Polydrug use and crime” members showed higher means and appears more maladjusted than “Alcohol only”. For instance, the factors that predicted both “Alcohol only”, “Polydrug use and crime” and/or “High crime” (parental supervision, sensation seeking behavior and conduct problems) had stronger association (weaker for parental supervision) to the latter groups. The latter groups also had higher levels of these risks than “Alcohol only”. “Polydrug use and crime” and “High crime” also consistently had higher level of risk among all factors that reached significance for “Alcohol only”, but not for the aforementioned classes. It has been emphasized previously that the most serious antisocial individual, such as the life course persistent offender in Moffit’s taxonomy, display both problematic individual and familial factors [53]. Factors that both “Polydrug use and crime” and “High crime” displayed high levels of. Risk factors also have a cumulative effect, where more factors present leads to a higher risk of an outcome [56]. The “Polydrug use and crime” and “High crime” classes could possibly also have a wider range of different risk factors present, leading to problematic behaviors. In order to examine if risk factor severity and accumulation contributes to these more maladapted classes, a larger sample is required.*” to the manuscript.*

Alcohol is a significant risk factor for criminal behavior; however, in the "Alcohol only" class of your sample, it did not co-occur with criminal behavior. A broader discussion is needed to explore the possible reasons for this discrepancy. Is it due to the way criminal behavior was operationalized, or are there other factors that are stronger predictors of criminal behavior at this particular developmental stage than alcohol consumption alone? A more comprehensive exploration of these factors would be valuable.

*Authors’ response: This is a very valid and interesting observation. We indeed agree that this requires further inquiry. Our interpretation and understanding of this result is, as you mentioned, that there are more factors at play that have a stronger implication on criminal behavior than just alcohol use. For instance, an experimental study has suggested that alcohol in itself does not cause a particular behavior in itself, but rather directs behaviors which are caused by other factor, in this study it was aggressive promoting or inhibiting cues. We also refer to that for instance in Moffit’s taxonomy the life course persistent offender’s (i.e. most frequent type of criminal offender) criminal activity is attributed to several pathological and environmental factors from a very early life stage. We want to emphasize though that in this study we did not test this and that it is speculative. At the end of the paragraph discussing the “alcohol only” class we have added “*This class also did not engage in any extensive delinquency despite having the highest alcohol consumption. This is notable since alcohol has been previously implied to be a contributing factor to criminal behavior, especially aggression and violence [14]. However, some research imply that alcohol use does not cause this behavior in itself, but rather directs it onto things such as provoking or inhibitory cues [52]. Also, it has been implied that the most frequent and persistent criminal offenders are driven by different familial and pathological factors early on in life, such as family problems, mental health issues and problematic personality [53]. It may be that there are other factors which are more driving in delinquency than only alcohol consumption., This could be possible, since judging by the mean scores of risk factors among the classes that engaged in substance use and/or delinquency, the “Alcohol only” class seemed to be the most well-adjusted. Though, it should be noted that this is not something that has been tested in this study and therefore remains speculative.*”.*

In addition, when describing the third class, you could be more specific by stating that adolescents in this group were more likely to consume a variety of substances, with the highest probability of cannabis use. They were also more likely to commit crimes compared to both the reference category and the second class, although the difference was not so big. The results should be discussed in this context to provide a clearer understanding of the factors at play.

*Authors’ response: We have added this explanation in the description of the third class to highlight this context. We have revised the description into “*Further, a “Polydrug use and crime” class was identified with co-occurrence of multiple substances, both alcohol, cannabis and illicit drugs, and acts of delinquency. This class had high alcohol consumption, though they were still consuming less than “Alcohol only”. This class also had the highest illicit drug use compared to the other classes, particularly cannabis. This class was also more likely to commit crime than both the “Low/Abstainers” class and the “Alcohol only” class. Though this difference was not as large as between the “High crime” class and the other classes.*”. We have also added “*Moreover, a “High crime” class was identified who scored the highest on all crime types, considerably higher for every crime type compared to “Polydrug use and crime” who was the second highest for crime among the classes*” to the description of the fourth class to clarify that “polydrug use and crime” was not as complicit in committing crime as “high crime”.*

The discussion of the fourth class is superficial and should be grounded in evidence and prior research.

*Authors’ response:* *We have attempted to make the discussion of the “High crime” class deeper and to tie it better to the existing literature. We tried to do this by drawing parallels to crime statistics in Sweden and also highlighting that some member of this class might developing a pattern similar to that of life course persistent offender in Moffit’s taxonomy. We have revised this part into “*Moreover, a “High crime” class was identified who scored the highest on all crime types, considerably higher for every crime type compared to “Polydrug use and crime” who was the second highest for crime among the classes. This class also engaged in co-occurring behaviors, namely consuming alcohol, illicit drugs and committing crimes. The alcohol consumption of the “High crime” class was also considerably higher than for the “Low/abstainers” class. Also, this class had the second highest illicit drug use. In the general population, the number of individuals who display a pattern of high frequent delinquent behaviors is often smaller [32]. It has also been established that a small group of individuals commit a disproportionate number of crimes in the general population. An example of this phenomenon is that 68% of violent crimes in Sweden can be attributed to 1% of the population [54]. Another example of this is that of the life course persistent offender in Moffit’s taxonomy. A group of offenders that is very small in size, but commit a disproportionately high frequency of criminal offences and also begin their offending in early adolescence and childhood [53]. This can correspond to the “High crime” class that was identified in this study – a very small group that committed the highest amount of crime. Though in this stage it is not clear how the further criminal behavior of this class will develop. In Moffit’s taxonomy the life course persistent offender differs from the adolescent limited offender who ceases their offending in adulthood. Some delinquent behavior during adolescence can be considered normative and the majority of these phase out in the years to follow [20, 53]. It is not possible to discern in this study which members of “High crime” class will cease their delinquency and who will retain it into adulthood. It could be that some of the members of this class will go on to develop a pattern of offending close to that of the life course persistent offender, but this is at this stage speculative.*“*

Considering the predictors of the classes, I suggest starting the discussion with the significant results, and then addressing the non-significant ones.

*Authors’ response: We have revised the order so that the significant ones now come first.*

Instead of saying 'considerably higher OR,' explain what it means.

*Authors’ response: The statements has been revised into* “had stronger association (weaker for parental supervision) to the latter groups.*”*

You should give more consideration to how measuring criminal behavior through self-report might influence your results.

*Authors’ response: We have added the sentences “*Another limitation refers to the use of self-report which relies on the recall of participants. There is also the risk of both under- and overreporting of crimes committed and substances consumed which might affect the reliability.*” to the strengths and limitations section to clarify the limitation of self-report.*

Based on the limitations and your findings, are there any recommendations for future research?

*Authors’ response: We have added a paragraph at the end of the limitations section where we highlight future directions we recommend. This paragraph reads “*With the inability to detect significant risk factors due to low prevalence of substance use and delinquency in the sample and limited statistical power, future studies should consider larger samples to reduce this problem. Another strategy for future research is to employ official registers such as medical records and crime registries. Since this study was biased towards those who did not engage in substance use and/or delinquency, official records could be possible way for studies with similar design to reach participants who could not be reached otherwise. Future studies might also want to consider comparing different types of substances and how the relate to and co-occur with delinquency. Also, longer time windows should be investigated which enables to examines developmental trajectories and differences between those whose substance use and delinquency is limited to adolescent years and those persist into later years*.”*

What is the added value of your study, and what are the potential clinical implications?

*Authors’ response: We have added a section on implications. We emphasize that for interventions, since the majority of individuals are not engaged in substance use or delinquency it may be that most individuals are not in need of any interventions. We also, emphasize that since it seems that the risk profile for substance use/delinquency consists of many different factors and thus interventions likely need to be multifactorial as well. The section we have added reads “*There are some implications with these results. Firstly, since there are implications that extensive substance use and delinquency seems to be limited to small part of the general population and most are not in need of any interventional actions. Interventions may be located and focused to where they are most needed instead. Though, this should be done with caution as targeted interventions may run the risk of labeling and stigmatizing individuals. Secondly, this study and research before indicate that the risk factor profile of the most severe substance users and delinquent offenders is multifactorial. The most extensive users and offenders have a high level and wide range of several different risk factors. It may be that interventions also need to be multifactorial and cover several different problem areas, such as improving mental health, family conditions, peer relations and school environment etc., in order to diminish and prevent further substance use and delinquent behaviors in the future.*”.*

Conclusion

The conclusion section should better summarize the main findings of the study and provide a clear take-home message.

*Authors’ response: We have revised the conclusion in an attempt to move away from repeating the results and better summarize the study. The conclusion now reads “*This study sought to identify different latent classes of substance misuse and delinquency in adolescence, and developmental risk factors associated with them. The vast majority of the participants do not engage in any extensive substance misuse or delinquency. The most common form of substance use was alcohol, likely adolescent binge drinking, and the most extensive substance use and delinquency was attributed about 3% of the sample. Substance users and delinquent offenders also show a multifactorial risk profile where several factors, such as familial problems, mental health mental health issues, personality traits, peer factors and other factors are related to belonging to a class with substance use and/or committing delinquency. This has implications that interventions might need to cover several factors as well. A relatively short time window has been investigated and future research should investigate longer time frames to establish prevalence of which limit their consumption/delinquency to adolescence and which persist into adult years. Statistical power was also a problem in this study and future research should use larger samples or other methods in an attempt to improve this.*”.*
